# A Machine Learning-Based Image Segmentation Method to Quantify In Vitro Osteoclast Culture Endpoints

**DOI:** 10.1007/s00223-023-01121-z

**Published:** 2023-08-11

**Authors:** Bethan K. Davies, Andrew P. Hibbert, Scott J. Roberts, Helen C. Roberts, Jennifer C. Tickner, Gill Holdsworth, Timothy R. Arnett, Isabel R. Orriss

**Affiliations:** 1 Department of Comparative Biomedical Sciences, Royal Veterinary College, Royal College Street, London, NW1 0TU UK; 2 Present Address: Clinical and Experimental Endocrinology, KU Leuven, Leuven, Belgium; 3 Department of Natural Sciences, Middlesex University, London, UK; 4 School of Pathology and Laboratory Medicine, University of Western Australia, Perth, Australia; 5 UCB Pharma, Slough, UK; 6 Department of Cell and Developmental Biology, University College London, London, UK

**Keywords:** Osteoclast, Formation, Resorption, Ilastik, Machine learning

## Abstract

Quantification of in vitro osteoclast cultures (e.g. cell number) often relies on manual counting methods. These approaches are labour intensive, time consuming and result in substantial inter- and intra-user variability. This study aimed to develop and validate an automated workflow to robustly quantify in vitro osteoclast cultures. Using ilastik, a machine learning-based image analysis software, images of tartrate resistant acid phosphatase-stained mouse osteoclasts cultured on dentine discs were used to train the ilastik-based algorithm. Assessment of algorithm training showed that osteoclast numbers strongly correlated between manual- and automatically quantified values (*r* = 0.87). Osteoclasts were consistently faithfully segmented by the model when visually compared to the original reflective light images. The ability of this method to detect changes in osteoclast number in response to different treatments was validated using zoledronate, ticagrelor, and co-culture with MCF7 breast cancer cells. Manual and automated counting methods detected a 70% reduction (*p* < 0.05) in osteoclast number, when cultured with 10 nM zoledronate and a dose-dependent decrease with 1–10 μM ticagrelor (*p* < 0.05). Co-culture with MCF7 cells increased osteoclast number by ≥ 50% irrespective of quantification method. Overall, an automated image segmentation and analysis workflow, which consistently and sensitively identified in vitro osteoclasts, was developed. Advantages of this workflow are (1) significantly reduction in user variability of endpoint measurements (93%) and analysis time (80%); (2) detection of osteoclasts cultured on different substrates from different species; and (3) easy to use and freely available to use along with tutorial resources.

**Supplementary Information:**

The online version contains supplementary material available at 10.1007/s00223-023-01121-z.

## Introduction

Osteoclasts are multinucleated cells of haematopoietic lineage that resorb bone. Osteoclasts are typically cultured in vitro on a variety of physiological (e.g. cortical bone slices, dentine discs) or non-physiological (e.g. calcium phosphate-coated plates, tissue culture plastic or glass) substrates for analysis of cellular physiology, morphology, and biochemical endpoints. Typical osteoclast parameters measured include tartrate resistant acid phosphatase (TRAP) positivity, number, and resorptive activity as well as multinuclearity (≥ 2 nuclei per cell) and actin ring/ruffled border formation [1–4]. Of these, number and resorption area provide valuable data about osteoclast formation and activity and have historically been manually quantified through image-processing softwares such as ImageJ [1]. Whilst this method enables user confirmation of individual osteoclasts and associated resorption events, it is time consuming, labour intensive and results in substantial intra- and inter-user variability. Thus, there is a clear need to develop an automated method that allows quick, easy, and accurate analysis of in vitro osteoclast cultures.

Attempts to automate in vitro endpoint analyses have been described but often rely on independent and sequential steps of (1) counting osteoclasts; (2) clearing cells from dentine/bone discs [1, 5–7]; and (3) separate measurement of the resorption area [8, 9]. These processes are time consuming and effectively destroy the experiment, preventing revisitation later (e.g. for imaging). Currently, the only attempt to simultaneously quantify osteoclasts and bone surface erosion has been performed on histological sections [10]. TrapHisto is an open-source software integrated into ImageJ that semi-automates histomorphometric analysis of static and dynamic bone turnover parameters, particularly resorption analysis [10]. Recent advances mean that new technologies such as machine learning (ML) can now be used to develop an automated workflow for in vitro osteoclast cultures. ML is an application of artificial intelligence (AI) where constructed mathematical models automatically learn from existing data to create an algorithm that produces accurate predictions from new observations without being explicitly programmed [11, 12]. Supervised ML, such as decision tree algorithms and random forest, requires labelled examples from training datasets. The algorithm learns from the labelled objects and generates a predictive model that accurately sorts new data objects into categories [11, 13, 14].

Application of ML methods has improved understanding and analysis efficiency of complex biological data and processes, especially in genomics, systems biology, and image analysis [11, 15, 16]. However, extensive computational and mathematical knowledge has historically been required to build such ML models, making their application to niche biological questions and processes difficult. The development of ilastik, a free, open-source supervised ML-based bio-image analysis software, has since enabled non-computationally proficient researchers to develop methodologies to rapidly execute complicated image analyses [17]. This user-friendly software contains pre-defined workflows that are adapted by the operator to create bespoke image analysis pipelines whilst completely shielding users from the mathematical and computational complexities required to build the random forest algorithm [14, 17, 18]. Some applications of ilastik include measuring neuronal nuclei and cell bodies and osteoblast differentiation from mesenchymal stem cells [19, 20].

Historically, automatically quantifying osteoclasts in vitro has proven challenging due to the non-uniform cell shape, size, and considerable spacing between nuclei and the cytoplasm of single osteoclasts [8, 21]. Four recent reports have built complex AI-based models to quantify TRAP+ or fluorescently labelled osteoclasts cultured on plastic but not bone or dentine [22–25]. Resorption parameters were not quantified in any of these models [22–25]. To date, ML, specifically ilastik, has not been applied to simultaneously measure osteoclast culture endpoints such as osteoclast number and resorption area for cells grown on physiologically relevant substrates. Therefore, the aim of this study was to develop and validate an automated image segmentation workflow in ilastik to reliably, and robustly quantify osteoclast number and resorption area in vitro.

## Materials and Methods

### Reagents

All tissue culture reagents were purchased from Life Technologies (Paisley, UK), and chemical reagents and MCF7 cells were purchased from Sigma-Aldrich (Poole, UK), unless otherwise stated.

### Animals

C57BL/6J mice (Charles River, UK) were group housed under standard conditions with free access to food and water. All animal procedures complied with the UK Animals (Scientific Procedures) Act 1986 and were reviewed and approved by the Royal Veterinary College Research Ethics Committee.

### Osteoclast Formation and Resorption Cultures

#### Mouse Osteoclasts

Osteoclast precursor cells were isolated from the long bones of ≥ 6-week-old mice as previously described [1]. Basal cell culture medium was Minimum Essential Medium supplemented with 10% FCS, 2 mM l-glutamine, 100 U/mL penicillin, 100 μg/mL streptomycin, and 0.25 μg/mL amphotericin (complete mixture abbreviated to MEM). In a 96-well tray, cells were seeded onto 5 mm dentine discs (10^6^ cells/disc) in MEM supplemented with 100 nM PGE_2_, 200 ng/mL M-CSF, and 3 ng/mL RANKL (R&D Systems Ltd, Abingdon, UK). After 24 h, discs containing adherent osteoclast precursors were transferred to 6-well trays (4 discs/well in 4 mL medium) with treatment conditions for the duration of culture. Osteoclasts were either treated with 10 nM zoledronate (or PBS-vehicle control), 1–10 μM ticagrelor (Tocris Bioscience, Abingdon, UK, or a dimethyl sulfoxide (DMSO)-vehicle control) or co-cultured with MCF7 breast cancer cells on insert plates (10,000 MCF7 cells per well of a 24-well plate with 1 dentine disc with adherent osteoclasts). Culture medium was acidified to pH 7.0 through addition of 10 MEq/L H^+^ (as HCl) for the final 48 h to activate osteoclasts to resorb. Dentine discs with adherent osteoclasts were fixed in 2.5% glutaraldehyde after 7–9 days of culture and stained for TRAP activity.

#### Human Osteoclasts

Human peripheral blood mononuclear cell (PBMC)-derived osteoclasts were isolated and cultured, as previously described [7, 26, 27], on dentine discs or tissue culture plastic prior to TRAP staining. All protocols were approved by University College London Ethics of Human Research Committee and the Institutional Review board of the Leuven University (ML6195). All work was performed in accordance with the ethical standards as laid down in the 1964 Declaration of Helsinki and its later amendments.

### Image Acquisition and Manual Quantification of In Vitro Osteoclast Cultures

TRAP-stained osteoclasts on dentine discs were imaged by reflective light microscopy at ×5 magnification using a DM400B upright microscope with samples illuminated by an EL6000 light source via a partial reflector. Images were acquired using a DFC550 colour camera through the Leica application suite/LAS-X v3.7 (all from Leica Microsystems, UK). Two images (1.3 MPix) were taken per disc and saved in TIFF format. All images were acquired with identical settings (saturation value of 50, a gain of 1, gamma of 0.6 and a field intensity of 100%) and exposure times (15–20 ms). Osteoclast number and the area resorbed per dentine disc (using a 16 × 12 grid overlay of 0.08 inches^2^ area per point, a total of ~ 576 points over a whole dentine disc) were assessed blind by dot-counting morphometry using ImageJ v1.51j8 [28], as previously described [1]. For resorption area quantification, the area of the dentine disc is approximately 19.2 mm^2^; thus, the area associated with each grid point is 0.034 mm^2^. Osteoclast number and resorption area of sample images (*n* = 12) were measured three times by user 1 at 1-year intervals to measure the intra-user coefficient of variation (intra-CV). The same images were measured twice by user 2 over a 3-year period to calculate the inter-user CV (inter-CV).

#### Algorithm Parameterisation and Training

**Fig. 1:**
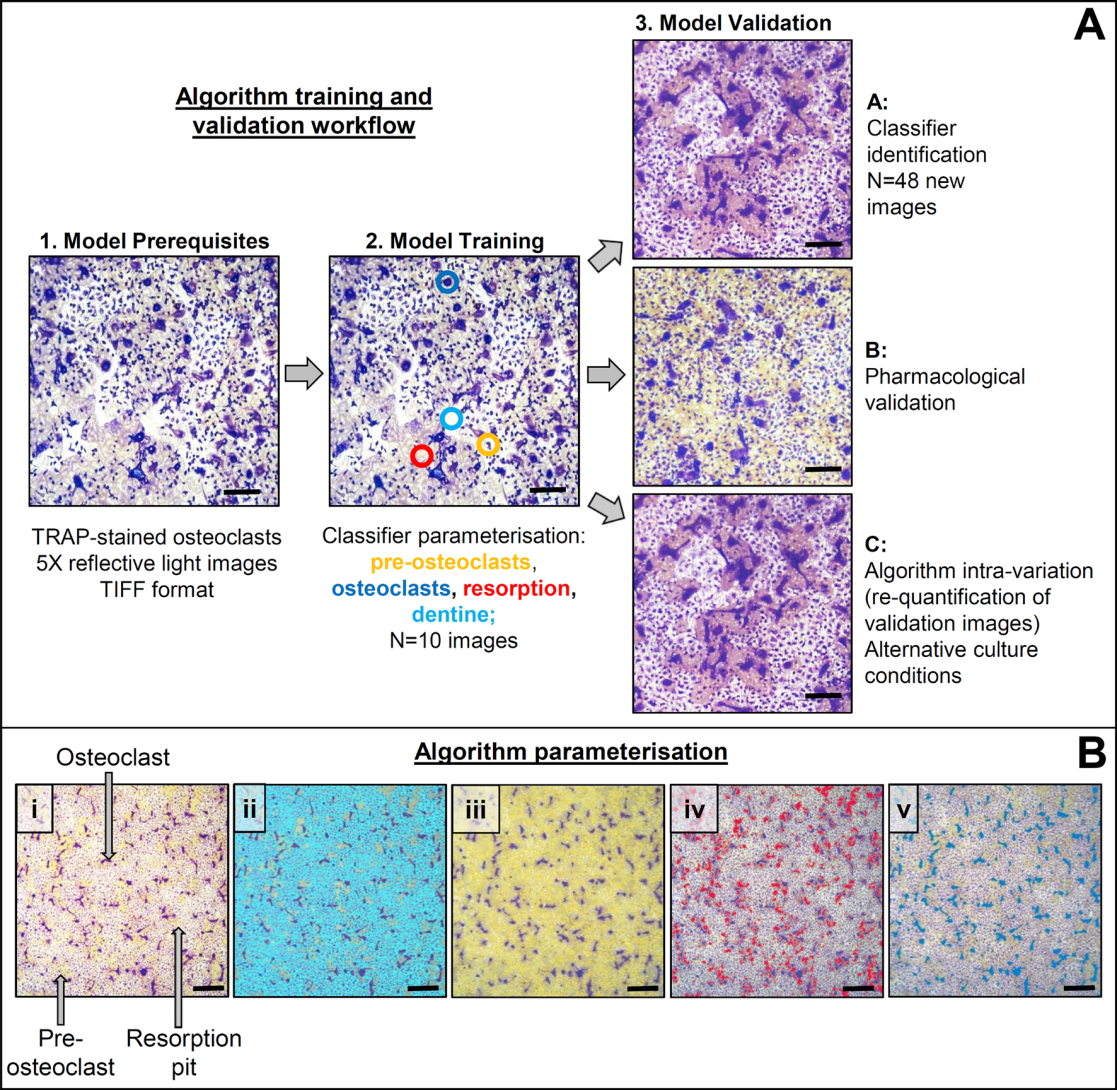
Developing, training, and validating the algorithm. **A** Training data consisting of (1) ×5 reflective light images of TRAP-stained osteoclasts were annotated within ilastik to identify osteoclasts, resorption events, pre-osteoclasts, and the dentine disc (2). Algorithm training was evaluated on new image sets (3A) prior to further validation on osteoclasts cultured with zoledronate, ticagrelor or MCF7 cells (3B). Finally, the intra-variability of model was determined by re-analysing previously quantified images, alteration of image orientation and measuring osteoclasts cultured from different species and/or on alternative substrates (3C). **B** Brushstroke annotation of the original reflective light image (i) in ilastik trained the random forest model to classify the dentine disc (light blue, ii), pre-osteoclasts (yellow, iii), resorption pits (red, iv), and osteoclasts (dark blue, v). Scale bar: 200 µm, *n* = 10

The pixel classification pipeline in ilastik v1.3.3 [17, 18] was used to generate an automated image segmentation of osteoclast culture endpoints. Figure 1A summarises the workflow employed to train and evaluate the ilastik algorithm. Ten reflective light images of TRAP-stained osteoclasts at various stages of differentiation and resorptive activity were selected as the training dataset. First, TIFF-formatted images were converted to the format file “.hdf5” (Hierarchical Data Format 5) using the ilastik plug-in in ImageJ [17] and loaded into ilastik. All available 2D pixel features (e.g. pixel colour, intensity, edge) across all given scales were included to train the ilastik model [17]. Pre-osteoclasts (smaller, uniformly shaped purple cells in Fig. 1), osteoclasts (larger, non-uniformly shaped purple cells, Fig. 1), resorption pits (tan areas surrounding osteoclasts, Fig. 1), and dentine disc (white background, Fig. 1) classifiers were identified in the training images by iterative brush strokes. The respective pixel features of these classifiers were computed by ilastik to segment images accordingly. It is important to note that users are completely shielded from the statistical and computational complexities of building the model. Ultimately, researchers without computational expertise can utilise ilastik for image analysis. Image segmentation predictions were assessed in real-time and additional annotations of images were made to correct erroneous categorisations. Once image segmentations were deemed appropriate (i.e. faithfully corresponded to the training image), the ilastik protocol was saved as the training file for subsequent validation and applied to new data without further supervision.

### Extraction of Quantitative Data from Automatically Segmented Images

**Fig. 2:**
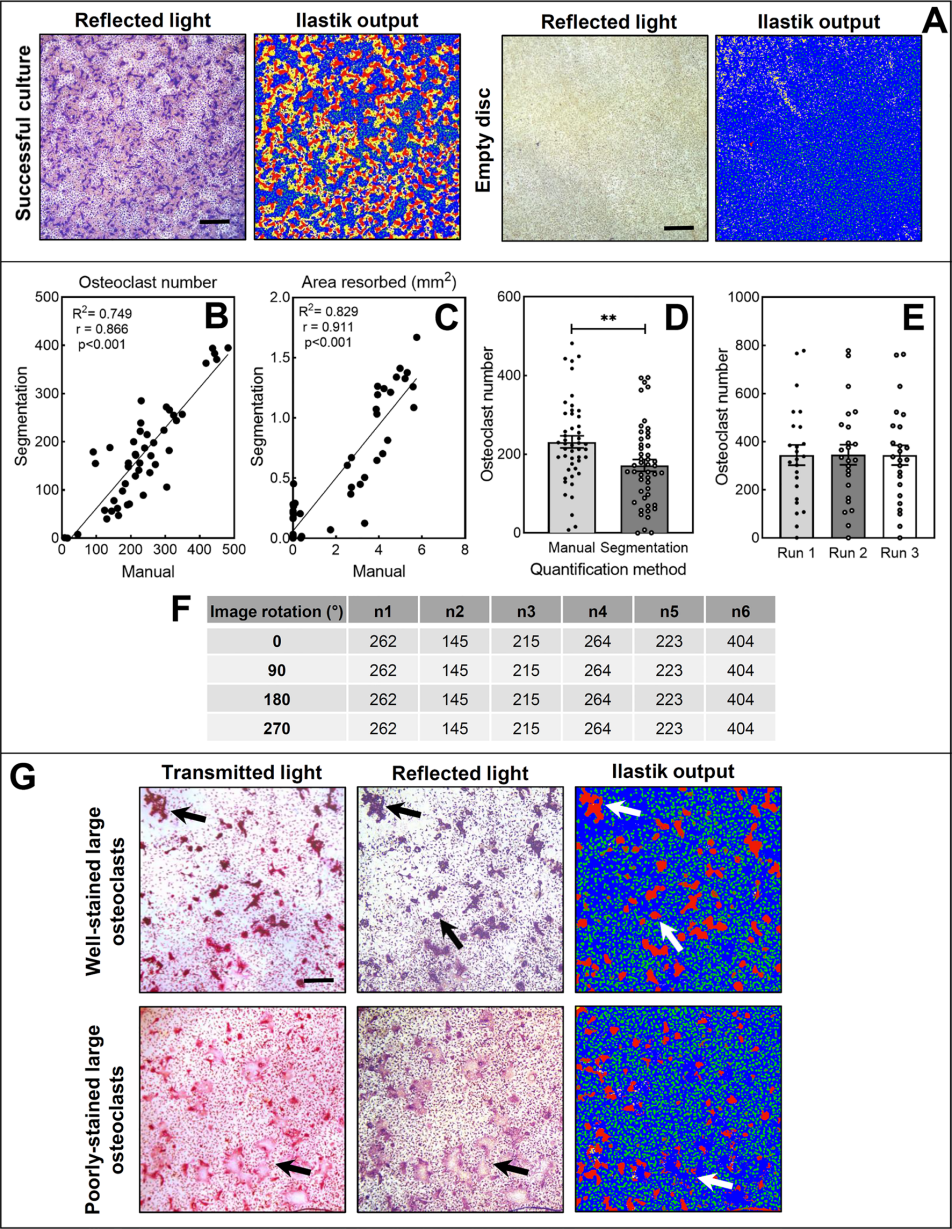
The ilastik model reliably detects and segments osteoclasts but not resorption events. **A** Osteoclasts (large purple cells) and resorption pits (tan areas) in the original reflective light image are segmented by ilastik (red = osteoclasts, yellow = resorption pits, green = pre-osteoclasts, blue = dentine disc). Images are representative of the typical segmentation output. Scale bar: 200 µm. The linear relationship between manual and automated quantification methods were assessed for osteoclast number (**B**) and the area resorbed (**C**). The Pearson correlation coefficient, *p* values, and line of best fit are shown. **D** Absolute osteoclast number was higher by manual quantification. Data presented as mean ± SEM with points for each training image (*n* = 48), **p < 0.01. Scale bar: 200 µm. **E** Re-running the same images through the algorithm over a 1-year period did not alter osteoclast number. **F** Rotating images at consecutive 90° angles does not affect automated osteoclast number quantification, *n* = 6. **G** Transmitted and reflective light images show that the model can detect osteoclasts of different sizes (illustrated by the black arrows in the microscopy images and the white arrows in the ilastik output). Uniform TRAP staining is required for appropriate segmentation of very large osteoclasts. Scale bar: 200 µm

**Fig. 3:**
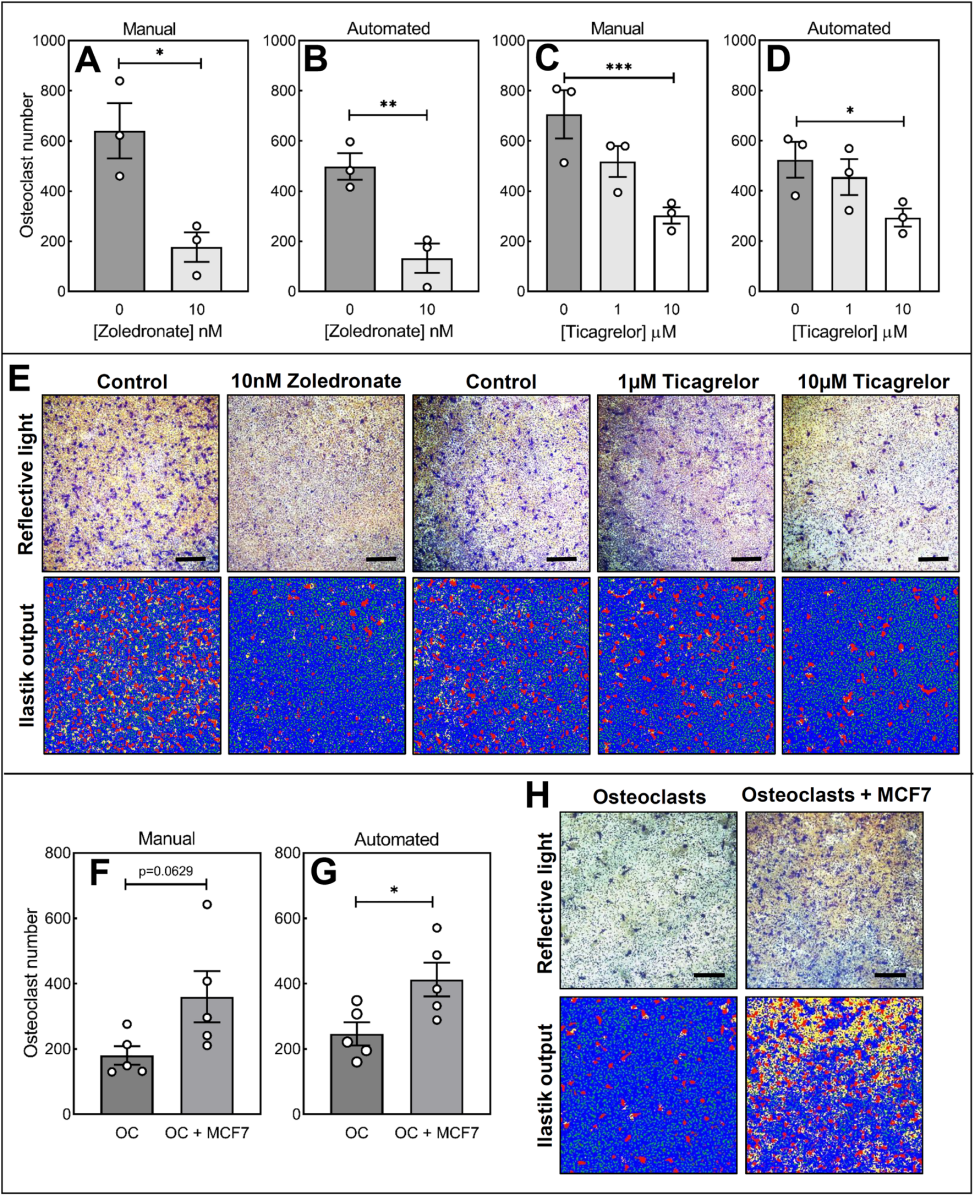
The ilastik model can detect biologically relevant increases and decreases in osteoclast number. The algorithm was pharmacologically validated using two agents with well-characterised inhibitory functions (zoledronate) or less well-characterised effects (ticagrelor). **A**, **B** 10 nM zoledronate and **C**, **D** 1–10 μM ticagrelor reduced osteoclast number by manual and automated quantification methods. **E** Irrespective of pharmacological agent used, the developed model faithfully segmented osteoclasts, but not resorption pits. Co-culture with MCF7 breast cancer cells increased osteoclast numbers as quantified through **F** manual and **G** automated methods. Data presented as mean ± SEM of 3–5 independent experiments, *p < 0.05, **p < 0.01 and ***p < 0.001. **H** Osteoclasts, but not resorption pits, were faithfully segmented from reflective light images (top row). For all ilastik images: bottom row, red = osteoclasts, yellow = resorption pits, green = pre-osteoclasts, blue = dentine disc). Scale bar: 200 µm

**Fig. 4:**
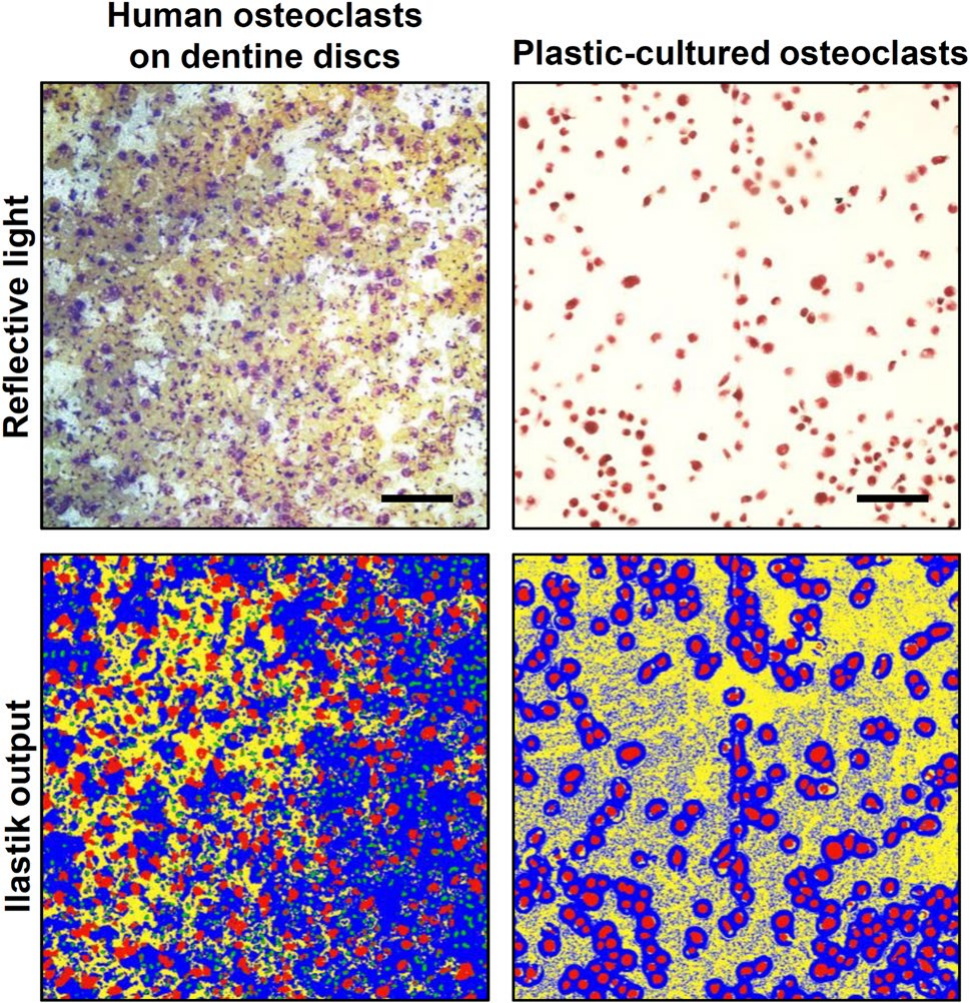
Human and plastic-cultured osteoclasts are detected by the ilastik model. Automated image segmentation identified human osteoclasts cultured on dentine discs and plastic-cultured osteoclasts compared to original reflective light images (top row). Representative images from 48 individual images, red = osteoclasts, yellow = resorption pits, green = pre-osteoclasts, blue = dentine disc. Scale: 200 µm

The trained ilastik model only outputs segmented images; therefore a FIJI, an image-processing package based on ImageJ [29], macro was written to combine the application of the automated segmentation workflow and extraction of quantitative features from each segmented image within the command line. The model and associated tutorials are freely available through this hyperlink: ILASTIK. Supp. Figure 1 summarises the macro-workflow and the user input required to run the model; this is the only information that users will need to enter to run the model. Briefly, raw ‘.TIFF’ images are first converted to ‘.hdf5’ format and imported into ilastik where the trained classifiers/model are applied to segment images. Segmented images are then exported. For ease of visualisation, the ‘Glow’ look-up table is applied to each segmented image presented in this paper, where each classifier is distinguishable by a particular colour. In this case, pre-osteoclasts are coloured green, osteoclasts are red, resorption events are yellow, and the dentine disc is blue (Figs. 2, 3, 4). This can be changed by users to suit their preferences or visual capacity. To extract the quantitative data, the image scale is set to 1 linear pixel equalling 2.031 µm (according to the spatial calibration of the microscope lens ×5 objective). The total area of each classifier within an image is subsequently calculated using the “Analyze particles” function in FIJI. A minimum osteoclast size threshold of 825 μm^2^ was determined using Volocity v6.3 (‘ThresholdBySize,’ PerkinElmer, Waltham, MA, USA) and applied to the osteoclast classifier to convert the area of osteoclasts per image to a discrete numerical value. Measurements are outputted in a “.csv” file where resorption area was converted to squared millimetres for comparison with manual values.

### Algorithm Validation

The validity of algorithm training was evaluated by processing unseen images from osteoclast cultures (*n* = 48). These same images were re-run through the algorithm three times over a 1-year period to establish its intra-variability. Images (*n* = 6) were rotated at sequential 90° angles in ImageJ and processed by the algorithm to establish whether image orientation alters pixel segmentation. Osteoclasts cultured with zoledronate, ticagrelor, or MCF7 cells were analysed to determine whether treatment effects could be detected by the automated method in the same way as manual quantification. Images from human osteoclast cultures were tested to establish if the algorithm could be used to quantify osteoclasts derived from different species and/or cultured on diverse substrates.

### Data Analysis

All data were presented and analysed using GraphPad Prism v9.3.1 (San Diego, USA). Data are presented as bar graphs with points to show values for individual experiments or box-and-whisker plots with min-to-max values. In vitro results show data from 3 to 5 individual experiments; each experiment was performed using osteoclasts isolated from different animals. Within each experiment, each group contained 6–8 technical replicates. Data were analysed using a two-tailed *t* test or randomised block ANOVA with Fisher’s LSD post hoc analysis [30]. During algorithm validation, values obtained from the automated segmentation were correlated with the corresponding manual quantification output. Both images of the same disc were kept independent (i.e. not summed) to observe individual trends. The Pearson correlation coefficient and simple linear regression analyses were calculated to determine the linear relationship between both methodologies.

## Results

### Variable Reproducibility of Manual Quantification of In Vitro Osteoclast Cultures

All three parameters (osteoclast number, total area resorbed, area resorbed per osteoclast) measured by user 1 (a PhD student) varied each year with an intra-coefficient of variation (intra-CV) of 22.1%, 22.3%, and 29.5%, respectively (Table 1). There were no differences in the measured parameters over time when measured by user 2 (an experienced researcher). The area resorbed per osteoclast varied between users (*p* < 0.05); measurements were 1.2-fold higher in user 1 than user 2 with an inter-CV of 3.3%. There were no differences in osteoclast number nor total area resorbed between operators (inter-CVs of 2.4% and 1.6%, respectively).

**Table 1:** Example user variation of manual osteoclast culture endpoint analysis

	Intra-variation (user 1, inexperienced researcher)				Intra-variation (user 2, experienced researcher)			Inter-variation (%)
	Year 1	Year 2	Year 3	Intra-CV (%)	Year 1	Year 3	Intra-CV (%)	
Osteoclast number	331 ± 89.1	232 ± 109.7^#^	265 ± 112.5^#$^	22.1	304.8 ± 106.8	296.7 ± 110	2.4	9.6
Total area resorbed (mm^2^)	10.0 ± 1.9	9.7 ± 2.0	6.7 ± 2.3^#$^	22.3	6.23 ± 2.19	6.24 ± 2.13	1.6	5.8
Area resorbed per osteoclast (mm^2^)	0.0308 ± 0.003	0.0458 ± 0.01^#^	0.0261 ± 0.003^#$^	29.5	0.0204 ± 0.001*	0.0212 ± 0.001*	3.3	14.6

The intra-user coefficient of variance (intra-CV) and inter-user coefficient of variance (inter-CV) of 12 images. Data presented as mean ± SD, *n* = 12 images

*CV* coefficient of variance

*Statistical difference (*p* < 0.05) from user 1’s year 3 measurements

^#^Statistical difference (*p* < 0.05) from user 1’s year 1 measurements

^$^Statistical difference (*p* < 0.01) from user 1’s year 2 measurements

### Ilastik Algorithm Parameterisation Faithfully Maps the Raw Training Image

During algorithm training, the key features, termed “classifiers,” in the reflective light images (Fig. 1Bi) were faithfully annotated using basic brushstrokes. The classifiers generated according to pixel features were the dentine disc (light blue, ii), pre-osteoclasts (yellow, iii), resorption pits (red, iv), and osteoclasts (dark blue, v).

### Segmented Images Produced by the Model are Faithful to the Original Image

Osteoclasts and pre-osteoclasts were faithfully segmented in images previously unseen by the model (Fig. 2). Osteoclast number and total area resorbed strongly correlated with manual quantification values (correlation coefficient, *r*, of 0.87 and 0.9, *p* < 0.001, respectively (Fig. 2B, C). Some resorption events were inaccurately segmented in discs considered to lack resorption pits by manual analysis (Fig. 2A). Absolute osteoclast number was 25% lower in the automated method than manually acquired values (*p* < 0.01, Fig. 2D). Algorithm re-training did not reduce the false identification of resorption pits (Supp. Fig. 2). Despite the strong positive correlation between manual and automated methods, consistent and accurate identification of resorption events proved challenging regardless of algorithm re-training. The developed model was, therefore, further validated to quantify osteoclast cell counts only.

The model can accurately detect osteoclasts of different sizes; however, for very large osteoclasts (≥ 100 μm) to be accurately segmented, uniform TRAP staining is required (Fig. 2G). Big osteoclasts that are unevenly stained are not as faithfully segmented as those that are fully TRAP stained (Fig. 2G).

### Osteoclast Numbers Acquired from the Model are Unaffected by Image Orientation

To investigate whether repeated analysis or image orientation impacted the output of the algorithm, images were re-analysed over a 1-year period and with a different image rotation, respectively. There were no differences in osteoclast number upon re-analysis over a 1-year period (Fig. 2E). Osteoclast number varied between individual discs and was reflective of the experimental conditions. This variability was faithfully recapitulated when re-analysed over a 1-year period. The model intra-CV for osteoclast number was 1.5%. Image orientation had no effect on the osteoclast number detected by the algorithm (Fig. 2F).

### Pharmacological and Physiological Validation of the Algorithm

The algorithm was validated by comparing the osteoclast numbers obtained from manual and automated analysis in osteoclasts exposed to: (1) a known inhibitor of osteoclast formation, the bisphosphonate zoledronate; (2) a less well-defined inhibitor of osteoclasts, the P2Y_12_ receptor antagonist ticagrelor; and (3) co-culture with MCF7 breast cancer cells, which are known to promote osteoclast formation [31, 32].

#### Zoledronate

Manual and automated methods respectively detected a 3.6-fold (*p* < 0.05) and 3.7-fold (*p* < 0.01) reduction in osteoclast number when treated with 10 nM zoledronate (Fig. 3A, B). Mature osteoclasts, pre-osteoclasts, and the dentine disc were faithfully segmented (Fig. 3E). Resorption events were not robustly detected by the automated method.

#### Ticagrelor

Both quantification methods detected a dose-dependent decrease in osteoclast number. Treatment with 10 μM ticagrelor caused a 57% (*p* < 0.001) and 60% (*p* < 0.05) reduction by manual and automated analysis, respectively (Fig. 3C, D). Automated image segmentation accurately detected osteoclasts, pre-osteoclasts, and the dentine disc, but not resorption area (Fig. 3E).

#### MCF7 Breast Cancer Cells

Co-culture with MCF7 breast cancer cells caused a 2-fold (*p* = 0.0629) and 1.6-fold (*p* < 0.05) increase in osteoclast number upon when quantified manually and automatically, respectively (Fig. 3F, G). Osteoclasts, pre-osteoclasts, and the dentine disc, but not resorption area, were faithfully segmented by the ilastik model (Fig. 3H).

### The Model can Detect and Quantify Human Osteoclasts Cultured on Dentine and Plastic

As the model was developed and validated on mouse bone marrow-derived osteoclasts, its flexibility to different species and culturing practices was also investigated. First, human osteoclasts cultured on dentine discs were reliably segmented by the automated method (Fig. 4). The identification of resorption events by the algorithm was inconsistent. Osteoclasts cultured on tissue culture plastic were also effectively identified by this method (Fig. 4). However, tissue culture plastic background was consistently and incorrectly identified as resorption events. Pixels were classified as dentine immediately surrounding the osteoclasts (as illustrated by the blue ring surrounding the cells on the ilastik output images).

## Discussion

In vitro cultures are widely used to study osteoclast biology. The unique nature of these cells means that analysis of osteoclast culture endpoints is typically performed manually and/or involves clearance of osteoclasts from the resorptive surface [7, 9]. However, these manual analysis methods are time consuming, labour intensive, and subjective. This work has utilised freely available software to develop and validate an automatic image segmentation workflow that enables quick, accurate, and reproducible quantification of in vitro osteoclast culture endpoints. The significant experimental advantages of this new method compared to established manual techniques are shown in Table 2.

**Table 2:** Advantages of using ilastik-based, automated osteoclast endpoint quantitative methods

	Manual	Ilastik
Intra-user variability	High	Very low
Inter-user variability	High	Very low
Training time/experience needed	Training 1–2 h	1 h to watch tutorials & install software
	Data produced is influenced by user experience	Data produced not influenced by user experience
Analysis time: osteoclast number	~ 5 min/disc	< 1 min/disc
	80 disc experiment = ~ 7 h researcher time	80 disc experiment = ~ 5 min researcher time to set up workflow then ~ 1 h automated analysis
Sequential number and resorption quantification	Yes, but time consuming	No, osteoclast number only
Suitable for use with cells grown on plastic and dentine	Yes	Yes
Applicable to different species	Yes	Yes

Ilastik, a ML-based imaging software, was trained to identify pre-osteoclasts, osteoclasts, resorption pits, and the dentine disc. Extensive testing revealed that the algorithm could accurately identify osteoclasts and distinguish between pre-osteoclasts and mature cells; however, detection of resorption pits was less reliable. To determine if this approach was sensitive enough to detect increases or decreases in osteoclast number, the algorithm was validated using two pharmacological agents and co-culture with MCF7 cells. Treatment with the bisphosphonate, zoledronate (10 nM), reduced osteoclast number, irrespective of quantification method used. This is consistent with previous reports that show an inhibitory effect of zoledronate on osteoclast number using manual quantification [33–35]. Second, osteoclasts were cultured with ticagrelor, a P2Y_12_ receptor antagonist typically used to inhibit platelet aggregation [36]. Dose-dependent decreases in osteoclast number were detected by both manual and automated methods. This is in line with an earlier study that also reported a ~ 60% reduction in osteoclasts at 10 μM ticagrelor [37]. Finally, an increase in osteoclast number was robustly detected by the ilastik model upon co-culture with MCF7 breast cancer cells. This is consistent with previous reports which show that MCF7 cells can promote osteoclastogenesis [31, 32]. Taken together, these findings suggest that the developed algorithm can be implemented to identify treatment effects (inhibitory or stimulatory), address biological questions and sensitively quantify subtle differences in osteoclast number.

Although accurate segmentation of bone marrow-derived mouse osteoclasts was achieved, absolute osteoclast number was usually lower than manually obtained values. The likely explanation for the absolute differences is the significant intra- and inter-variation in manually quantified values by operators, preventing the establishment of ground truth. Ground truth is a set of measurements that are known to be accurate and is used to assess the precision of a developed ML model. Operator variability is rarely reported within the literature despite manual quantification being the gold standard for measuring osteoclast parameters in vitro. In histomorphometric analyses, Tong et al. reported manual variability of ≥ 50% when analysing the same histological sample on six different occasions even with strictly defined parameters [38]. In the current study, intra-variation was assessed across 2 users by quantifying the same discs over 2–3 consecutive years. Significant differences in the osteoclast number obtained were observed in user 1 (a PhD student with no prior experience quantifying osteoclast culture endpoints), but not user 2 (an established researcher with > 20 years’ experience of manual osteoclast quantification). This suggests that user experience is likely a major factor influencing variability. Similarly, minor image modifications (e.g. brightness and contrast) to better visualise osteoclasts and resorption pits during manual analysis may also contribute to user variation. Despite differences in absolute osteoclast number, similar trends were reported between users. Consequently, the accuracy of the trained model was estimated by qualitative assessment of segmented images and comparing treatment responses, rather than absolute numbers, between both quantification methods.

The ilastik algorithm variance is 1.5% and represents a 93% reduction in user variability for osteoclast number compared to the manual method (Table 2). Furthermore, no differences in osteoclast number were recorded upon re-analysis of the same image sets and irrespective of image orientation. This highlights the robustness and reliability of this new automated osteoclast quantification method which can also reduce the inherent analysis variability posed by inexperienced users. Similar reductions in user variability upon automation of histomorphometric analyses have been reported [10, 39–41]. In contrast, the recent AI-based models quantifying in vitro osteoclasts on plastic did not measure improvements in operator variability from manual counting methods [22–25]. The ilastik model presented in this study requires limited operator input of defined parameters (as defined in Supp. Fig. 1B) for image segmentation and no algorithm re-training prior to implementation, further limiting the introduction of user variation. It should, however, be noted that variability could be introduced should users alter the original training file, image scale, or osteoclast size threshold from what has been described and optimised. Furthermore, image quality (e.g. brightness, staining) can impact osteoclast quantification. For example, homogenous TRAP staining is essential for accurate image segmentation, particularly when quantifying larger osteoclasts. Alterations to the pixel features (e.g. colour, brightness, texture, edge) modify the random forest decision surface in ilastik for classifier categorisation [17] which impacts the accuracy of the model. Consequently, image settings were optimised here to ensure appropriate segmentation of classifiers including a defined exposure time range, saturation and gain that are applicable across all images and users.

Overall, this user-friendly ilastik model shows that simple microscopy and staining can be used to robustly detect osteoclasts from different species (mouse and human), sample illumination (reflective light and brightfield) and seeding substrate (dentine disc and plastic) without additional re-training of the model. Furthermore, this pipeline reduces analysis time by 80% whereby osteoclast number from 1 disc is obtained in ~ 1 min compared to ~ 5 min when counted manually. Recently, Cohen-Karlik et al. trained a deep ML algorithm by manually contouring each cell cultured on plastic to classify TRAP-stained pre-osteoclasts, mature osteoclasts (3–14 nuclei) and hyper-nucleated osteoclasts (≥ 15 nuclei) [22]. Alternatively, Maurin et al. fluorescently labelled nuclei, F-actin, and microtubules and used CellProfiler™ to automatically segment primary osteoclasts cultured on tissue culture plastic [23]. However, unlike ilastik, these pipelines are time consuming and reliant on extensive and complex mathematical and computational knowledge for their manual construction and subsequent re-training for individual operators’ pipelines. In contrast, our model is quick, easy-to-use, flexible and readily implementable (with associated tutorial resources) without any need of classifier re-training or mathematical and programming knowledge. This represents one of the main advantages of this algorithm over other previously reported automated models.

Whilst this model is very effective at measuring osteoclast number, further work is necessary to incorporate the unique features of osteoclasts (e.g. multinucleation, actin ring) into an ilastik workflow for in vitro endpoint analysis. For example, although TRAP staining is an excellent way of staining osteoclasts, using it to visualise nuclei is more problematic, primarily because it is very easy to overstain cells. Thus, an alternative staining approach similar to Maurin et al. [23] would be required to identify and quantify the number of nuclei per osteoclasts. However, if a new staining method was used, an entirely new ilastik model would need to be generated, trained and validated.

It is important to emphasise that this ilastik-based model has been optimised for in vitro osteoclast cultures, particularly dentine-cultured osteoclasts. Therefore, the algorithm parameterisation and training required to develop this method is specific to these conditions. Although plastic-cultured osteoclasts can be detected by the model, we advise that segmented images are reviewed for erroneous classification as the model has not been specifically trained and optimised to identify plastic-cultured osteoclasts. Furthermore, this model is not readily transferrable to other workflows where osteoclast quantification is needed (e.g. histology, histomorphometry). In principle, this software can be used to construct a new ilastik-based model for analysis of tissue sections.

Although the automated segmentation of osteoclasts was successful, accurately detecting resorption events proved challenging. Resorption pits were reliably identified in training but not during validation of image sets, suggesting that this classifier may be overfitted. Overfitting refers to over-specific training of the algorithm that minimises its generalised predictive power when exposed to new data. Whilst ilastik operates on minimal brushstroke annotations to train classifiers, it was necessary to add more brushstrokes to differentiate the pixel features at the resorption pit-dentine disc boundary. Similar difficulties assessing the resorption boundary have been previously reported [42]. Furthermore, the inherent variation between primary cultures, TRAP staining and the heterogeneity of the dentine disc surface hinders the determination of optimal pixel features that can be generalisable. Thus, providing more example images to train the ilastik model would be unlikely to improve the sensitivity of resorption pit delimitation. Use of a grid overlay to manually quantify resorption area remains the gold standard, but grid size and area are seldom reported leading to operator variability across research centres [43–45]. Semi-automatic methods are available to analyse resorption area but require the removal of cells from the discs, effectively destroying the experiment, and still introduces user variability [9, 10, 42]. It is, therefore, likely that more complex models, such as deep learning (DL), will be required to fully automate the simultaneous quantification of both osteoclast number and resorptive activity. DL has already successfully quantified osteoclast and nuclei numbers [22, 24, 25], but not resorption events. Due to greater processing layers, DL could discover complicated feature patterns in large datasets that better delimit the resorption pit-dentine disc boundary for osteoclast activity analysis.

In conclusion, a ML-based image segmentation workflow successfully identified mature osteoclasts, but not resorption events, and significantly reduced user variability and analysis time of in vitro endpoint quantification by 93% and 80%, respectively. This protocol is flexible to deviations in experimental set-up and can be readily implemented for standardised osteoclast quantification across skeletal research centres. The model and associated tutorials are freely available and readily implementable without any additional training or coding knowledge through this hyperlink: ILASTIK. Please contact the corresponding author if there are any issues accessing the files or if there are further questions.

### Supplementary Information

Below is the link to the electronic supplementary material.

Supplementary file1 (TIF 3810 KB)
**Supplementary Figure 1** Running the ilastik model to automate osteoclast endpoint quantification. **A** Prerequisites to running the model are TRAP-stained mouse osteoclasts imaged at ×5 magnification by reflective light microscopy saved in ‘.TIFF’ file formats. TIFF images are converted to HDF5 format, imported into ilastik where the trained classifiers are applied to segment images. Segmented images are exported to the specified file directory, where a look-up table can be applied to distinguish between classifiers. To extract the quantitative data, the image scale is set to 1 linear pixel equalling 2.031 µm. The total area of each classifier within an image is subsequently calculated using the “Analyze particles” function in FIJI. A minimum osteoclast size threshold of 825 μm^2^ is applied to the osteoclast classifier to convert the area of osteoclasts per image to a discrete numerical value. Measurements are outputted in a “.csv” file. **B** The ilastik model is incorporated within an automated FIJI macro/script with no graphical capabilities. The dialogue box requests the training file and classifier names, image scale and export style and size thresholds to be applied to classifiers to obtain absolute values. Each time the model is run this information only needs to be entered once and is the only user input required.

Supplementary file2 (TIF 9190 KB)
**Supplementary Figure 2** Algorithm re-training does not improve segmentation of resorption events. Osteoclasts (**A**) and the area resorbed (**B**) were either manually quantified or classified following re-training of the ilastik model. No differences in absolute osteoclast numbers and total area resorbed were observed between the original and re-trained ilastik model. Data presented as mean ± SEM with points for each training image (*n* = 48). **C** Osteoclasts (large purple cells) and resorption pits (tan areas) in the original image appear to be somewhat faithfully represented in the automatically segmented ilastik output (red = osteoclasts, yellow = resorption pits, green = preosteoclasts, blue = dentine disc). The ilastik output incorrectly segments resorption events despite the absence of resorption on the dentine disc and algorithm re-training (bottom row, zoomed in). Scale: 200 µm. Images are representative of the typical segmentation output.

## References

[1] Orriss IR, Arnett TR (2012). Rodent osteoclast cultures. Methods Mol Biol.

[2] Merrild DM, Pirapaharan DC, Andreasen CM, Kjærsgaard-Andersen P, Møller AM, Ding M (2015). Pit- and trench-forming osteoclasts: a distinction that matters. Bone Res.

[3] Piper K, Boyde A, Jones SJ (1992). The relationship between the number of nuclei of an osteoclast and its resorptive capability in vitro. Anat Embryol.

[4] Lees RL, Heersche JN (1999). Macrophage colony stimulating factor increases bone resorption in dispersed osteoclast cultures by increasing osteoclast size. J Bone Miner Res.

[5] Rumpler M, Würger T, Roschger P, Zwettler E, Sturmlechner I, Altmann P (2013). Osteoclasts on bone and dentin in vitro: mechanism of trail formation and comparison of resorption behavior. Calcif Tissue Int.

[6] Kleinhans C, Schmid FF, Schmid FV, Kluger PJ (2015). Comparison of osteoclastogenesis and resorption activity of human osteoclasts on tissue culture polystyrene and on natural extracellular bone matrix in 2D and 3D. J Biotechnol.

[7] Owen HC, Vanhees I, Solie L, Roberts SJ, Wauters A, Luyten FP (2012). Critical illness-related bone loss is associated with osteoclastic and angiogenic abnormalities. J Bone Miner Res.

[8] Kopesky P, Tiedemann K, Alkekhia D, Zechner C, Millard B, Schoeberl B (2014). Autocrine signaling is a key regulatory element during osteoclastogenesis. Biol Open.

[9] Itzstein C, van 't Hof RJ (2012). Osteoclast formation in mouse co-cultures. Methods Mol Biol.

[10] van ‘t Hof RJ, Rose L, Bassonga E, Daroszewska A (2017). Open source software for semi-automated histomorphometry of bone resorption and formation parameters. Bone.

[11] Tarca AL, Carey VJ, Chen X-w, Romero R, Drăghici S (2007). Machine learning and its applications to biology. PLoS Comput Biol.

[12] Lundervold AS, Lundervold A (2019). An overview of deep learning in medical imaging focusing on MRI. Z Med Phys.

[13] Geurts P, Irrthum A, Wehenkel L (2009). Supervised learning with decision tree-based methods in computational and systems biology. Mol Biosyst.

[14] Breiman L (2001). Random forests. Mach Learn.

[15] Han H, Liu W (2019). The coming era of artificial intelligence in biological data science. BMC Bioinform.

[16] Moen E, Bannon D, Kudo T, Graf W, Covert M, Van Valen D (2019). Deep learning for cellular image analysis. Nat Methods.

[17] Berg S, Kutra D, Kroeger T, Straehle CN, Kausler BX, Haubold C (2019). Ilastik: interactive machine learning for (bio)image analysis. Nat Methods.

[18] Sommer C, Straehle CN, Kothe U, Hamprecht FA (2011) Ilastik: interactive learning and segmentation toolkit. In: Eighth IEEE international symposium on biomedical imaging (ISBI) proceedings. pp 230–233

[19] Holcomb PS, Morehead M, Doretto G, Chen P, Berg S, Plaza S, Spirou G (2016). Rapid and semi-automated extraction of neuronal cell bodies and nuclei from electron microscopy image stacks. Methods Mol Biol.

[20] Bongiorno T, Kazlow J, Mezencev R, Griffiths S, Olivares-Navarrete R, McDonald JF (2014). Mechanical stiffness as an improved single-cell indicator of osteoblastic human mesenchymal stem cell differentiation. J Biomech.

[21] Millard BL, Niepel M, Menden MP, Muhlich JL, Sorger PK (2011). Adaptive informatics for multifactorial and high-content biological data. Nat Methods.

[22] Cohen-Karlik E, Awida Z, Bergman A, Eshed S, Nestor O, Kadashev M (2021). Quantification of osteoclasts in culture, powered by machine learning. Front Cell Dev Biol.

[23] Maurin J, Morel A, Hassen-Khodja C, Vives V, Jurdic P, Machuca-Gayet I (2018). Combined strategy of siRNA and osteoclast actin cytoskeleton automated imaging to identify novel regulators of bone resorption shows a non-mitotic function for anillin. Euro J Cell Biol.

[24] Wang X, Kittaka M, He Y, Zhang Y, Ueki Y, Kihara D (2022). OC_Finder: osteoclast segmentation, counting, and classification using watershed and deep learning. Front Bioinform.

[25] Kohtala S, Nedal TMV, Kriesi C, Moen SH, Ma Q, Ødegaard KS (2022). Automated quantification of human osteoclasts using object detection. Front Cell Dev Biol.

[26] Utting JC, Flanagan AM, Brandao-Burch A, Orriss IR, Arnett TR (2010). Hypoxia stimulates osteoclast formation from human peripheral blood. Cell Biochem Funct.

[27] Owen HC, Vanhees I, Gunst J, Van Cromphaut S, Van den Berghe G (2015). Critical illness-induced bone loss is related to deficient autophagy and histone hypomethylation. Intensive Care Med Exp.

[28] Schneider CA, Rasband WS, Eliceiri KW (2012). NIH Image to ImageJ: 25 years of image analysis. Nat Methods.

[29] Schindelin J, Arganda-Carreras I, Frise E, Kaynig V, Longair M, Pietzsch T (2012). Fiji: an open-source platform for biological-image analysis. Nat Methods.

[30] Festing MF (2001). Guidelines for the design and statistical analysis of experiments in papers submitted to ATLA. Altern Lab Anim.

[31] Nicolin V, Bortul R, Bareggi R, Baldini G, Martinelli B, Narducci P (2008). Breast adenocarcinoma MCF-7 cell line induces spontaneous osteoclastogenesis via a RANK-ligand-dependent pathway. Acta Histochem.

[32] Feng Q, Wang D, Feng J, Guo P, Geng C (2020). Denosumab inhibits MCF-7 cell line-induced spontaneous osteoclastogenesis via the RANKL/MALAT1/miR-124 axis. Transl Cancer Res.

[33] Li P, Zhao Z, Wang L, Jin X, Shen Y, Nan C (2018). Minimally effective concentration of zoledronic acid to suppress osteoclasts in vitro. Exp Ther Med.

[34] Huang X-L, Huang L-Y, Cheng Y-T, Li F, Zhou Q, Wu C (2019). Zoledronic acid inhibits osteoclast differentiation and function through the regulation of NF-κB and JNK signalling pathways. Int J Mol Med.

[35] Sims NA, Green JR, Glatt M, Schlict S, Martin TJ, Gillespie MT (2004). Targeting osteoclasts with zoledronic acid prevents bone destruction in collagen-induced arthritis. Arth Rheum.

[36] Wijns W, Kolh P, Danchin N, Di Mario C, Falk V, Folliguet T (2010). Guidelines on myocardial revascularization: the task force on myocardial revascularization of the European Society of Cardiology (ESC) and the European Association for Cardio-Thoracic Surgery (EACTS). Euro Heart J.

[37] Mediero A, Wilder T, Reddy VSR, Cheng Q, Tovar N, Coelho PG (2016). Ticagrelor regulates osteoblast and osteoclast function and promotes bone formation in vivo via an adenosine-dependent mechanism. FASEB J.

[38] Tong X-Y, Malo M, Tamminen IS, Isaksson H, Jurvelin JS, Kröger H (2015). Development of new criteria for cortical bone histomorphometry in femoral neck: intra- and inter-observer reproducibility. J Bone Miner Metab.

[39] Emmanuel T, Brüel A, Thomsen JS, Steiniche T, Brent MB (2012). Artificial intelligence-assisted identification and quantification of osteoclasts. MethodsX.

[40] Arganda-Carreras I, Kaynig V, Rueden C, Eliceiri KW, Schindelin J, Cardona A (2017). Trainable Weka Segmentation: a machine learning tool for microscopy pixel classification. Bioinformatics.

[41] Malhan D, Muelke M, Rosch S, Schaefer AB, Merboth F, Weisweiler D (2018). An optimized approach to perform bone histomorphometry. Front Endocrinol.

[42] Juvin R, Phelip X, Camus E, Usson Y (1990). An automatic method for bone histomorphometry: assessment with reference to usual static and dynamic parameters. J Bone Miner Res.

[43] Neutzsky-Wulff AV, Sørensen MG, Kocijancic D, Leeming DJ, Dziegiel MH, Karsdal MA (2010). Alterations in osteoclast function and phenotype induced by different inhibitors of bone resorption-implications for osteoclast quality. BMC Musculoskelet Disord.

[44] Karsdal MA, Hjorth P, Henriksen K, Kirkegaard T, Nielsen KL, Lou H (2003). Transforming growth factor-β controls human osteoclastogenesis through the p38 MAPK and regulation of RANK expression. J Biol Chem.

[45] Henriksen K, Gram J, Schaller S, Dahl BH, Dziegiel MH, Bollerslev J (2004). Characterization of osteoclasts from patients harboring a G215R mutation in ClC-7 causing autosomal dominant osteopetrosis type II. Am J Pathol.

