# Supplementary figures and images for: A Machine Learning-Based Image Segmentation Method to Quantify In Vitro Osteoclast Culture Endpoints
Source: Calcif Tissue Int. 2023 Aug 11;113(4):437–48. doi: 10.1007/s00223-023-01121-z (PMC10516805; doi:10.1007/s00223-023-01121-z)

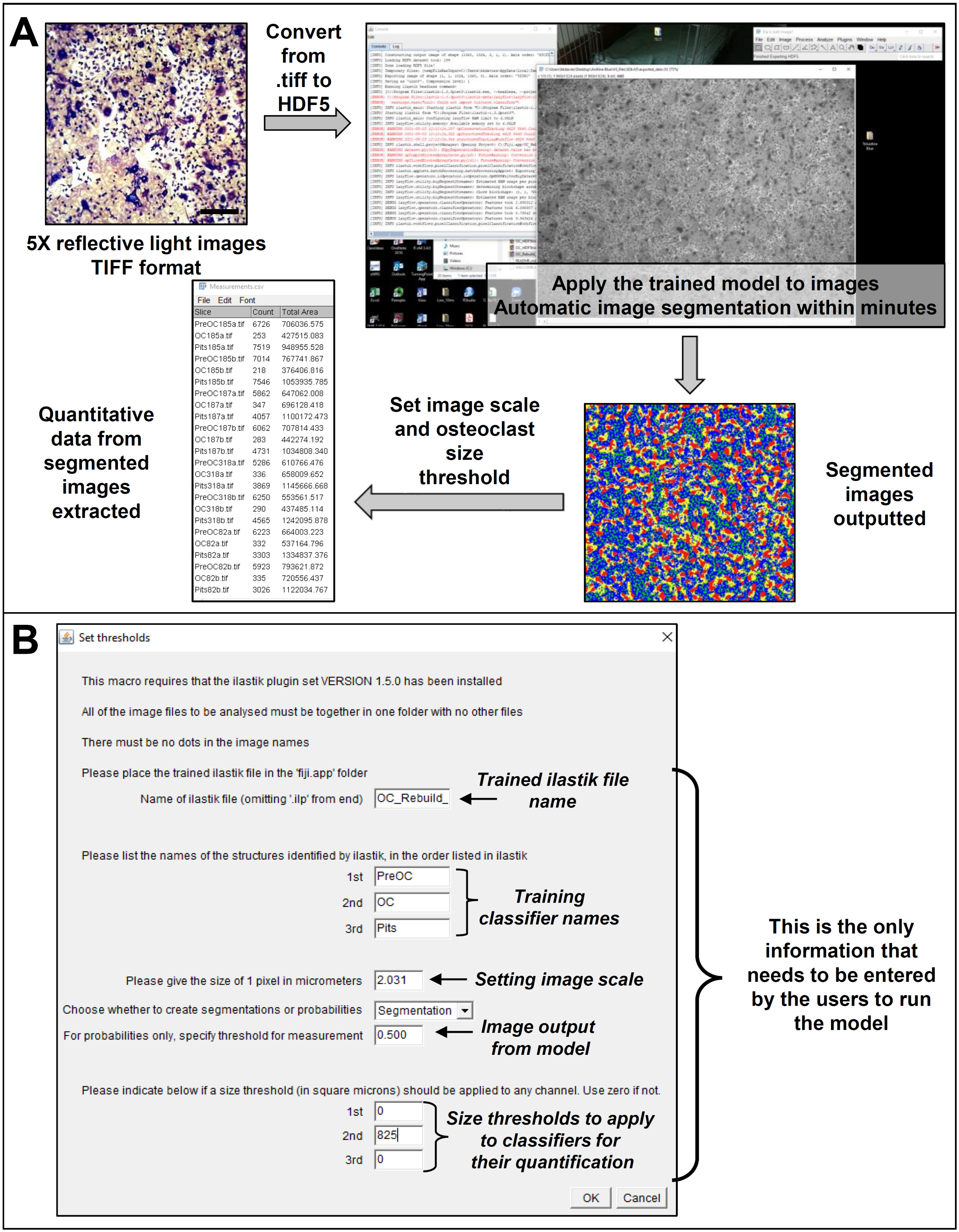

Supplement: Supplementary file 1 — Supplementary file1 (TIF 3810 KB) Supplementary Figure 1 Running the ilastik model to automate osteoclast endpoint quantification. A Prerequisites to running the model are TRAP-stained mouse osteoclasts imaged at ×5 magnification by reflective light microscopy saved in ‘.TIFF’ file formats. TIFF images are converted to HDF5 format, imported into ilastik where the trained classifiers are applied to segment images. Segmented images are exported to the specified file directory, where a look-up table can be applied to distinguish between classifiers. To extract the quantitative data, the image scale is set to 1 linear pixel equalling 2.031 µm. The total area of each classifier within an image is subsequently calculated using the “Analyze particles” function in FIJI. A minimum osteoclast size threshold of 825 μm2 is applied to the osteoclast classifier to convert the area of osteoclasts per image to a discrete numerical value. Measurements are outputted in a “.csv” file. B The ilastik model is incorporated within an automated FIJI macro/script with no graphical capabilities. The dialogue box requests the training file and classifier names, image scale and export style and size thresholds to be applied to classifiers to obtain absolute values. Each time the model is run this information only needs to be entered once and is the only user input required. [file 223_2023_1121_MOESM1_ESM.tif]

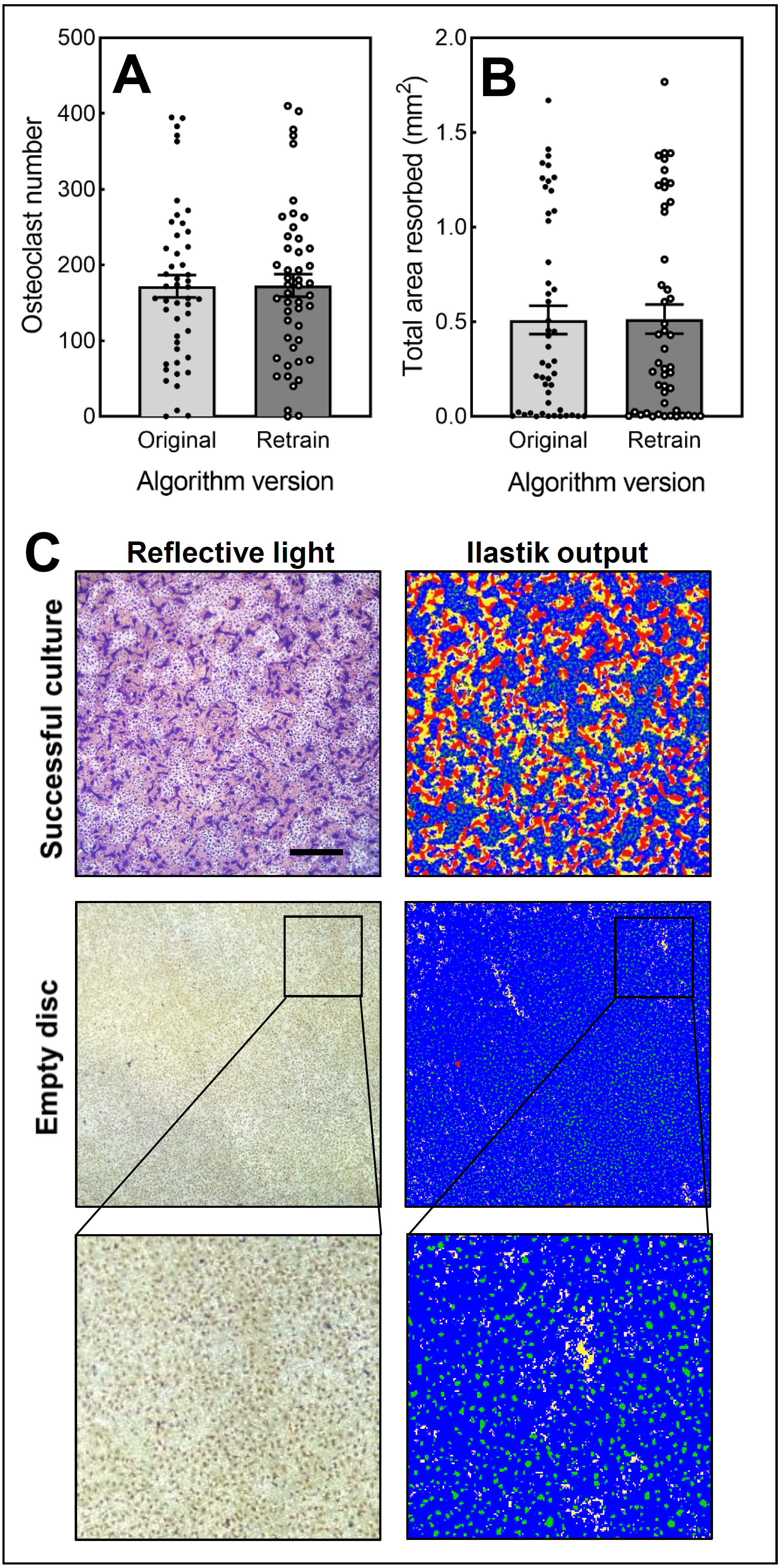

Supplement: Supplementary file 2 — Supplementary file2 (TIF 9190 KB) Supplementary Figure 2 Algorithm re-training does not improve segmentation of resorption events. Osteoclasts (A) and the area resorbed (B) were either manually quantified or classified following re-training of the ilastik model. No differences in absolute osteoclast numbers and total area resorbed were observed between the original and re-trained ilastik model. Data presented as mean ± SEM with points for each training image (n = 48). C Osteoclasts (large purple cells) and resorption pits (tan areas) in the original image appear to be somewhat faithfully represented in the automatically segmented ilastik output (red = osteoclasts, yellow = resorption pits, green = preosteoclasts, blue = dentine disc). The ilastik output incorrectly segments resorption events despite the absence of resorption on the dentine disc and algorithm re-training (bottom row, zoomed in). Scale: 200 µm. Images are representative of the typical segmentation output. [file 223_2023_1121_MOESM2_ESM.tif]
